# Unraveling the Genetic and Environmental Relationship Between Well-Being and Depressive Symptoms Throughout the Lifespan

**DOI:** 10.3389/fpsyt.2018.00261

**Published:** 2018-06-14

**Authors:** Bart M. L. Baselmans, Yayouk E. Willems, C. E. M. van Beijsterveldt, Lannie Ligthart, Gonneke Willemsen, Conor V. Dolan, Dorret I. Boomsma, Meike Bartels

**Affiliations:** ^1^Department of Biological Psychology, Vrije Universiteit Amsterdam, Amsterdam, Netherlands; ^2^Amsterdam Public Health Research Institute, Vrije Universiteit Amsterdam, Amsterdam, Netherlands; ^3^Neuroscience Amsterdam, Amsterdam, Netherlands

**Keywords:** well-being, depressive symptoms, heritability, childhood, adolescence, adulthood

## Abstract

Whether well-being and depressive symptoms can be considered as two sides of the same coin is widely debated. The aim of this study was to gain insight into the etiology of the association between well-being and depressive symptoms across the lifespan. In a large twin-design, including data from 43,427 twins between age 7 and 99, we estimated the association between well-being and depressive symptoms throughout the lifespan and assessed genetic and environmental contributions to the observed overlap. For both well-being (range 31–47%) and depressive symptoms (range 49–61%), genetic factors explained a substantial part of the phenotypic variance across the lifespan. Phenotypic correlations between well-being and depressive symptoms across ages ranged from −0.34 in childhood to −0.49 in adulthood. In children, genetic effects explained 49% of the phenotypic correlation while in adolescents and young adults, genetic effects explained 60–77% of the phenotypic correlations. Moderate to high genetic correlations (ranging from −0.59 to −0.66) were observed in adolescence and adulthood, while in childhood environmental correlations were substantial but genetic correlations small. Our results suggest that in childhood genetic and environmental effects are about equally important in explaining the relationship between well-being and depressive symptoms. From adolescence onwards, the role of genetic effects increases compared to environmental effects. These results provided more insights into the etiological underpinnings of well-being and depressive symptoms, possibly allowing to articulate better strategies for health promotion and resource allocation in the future.

## Introduction

Well-being plays an important role in scientific disciplines such as psychology, medicine, and public health policy (1–3). Also, well-being is a topic of great interest in disciplines such as economics with increasing numbers of studies exploring the link between economic factors and well-being [e.g., “whether money buys happiness”, see (4, 5)]. In contemporary sciences, well-being is often defined by a continuous spectrum of positive feelings and subjective life assessments, which is in line with the description of Diener et al. (6), who explained well-being as a broad category of phenomena that includes people's emotional responses, and global judgments of life satisfaction. A considerable number of studies show that well-being is positively associated with physical health (7), success (8), and longevity (3, 9). Additionally, well-being is associated with less mental illness, notably mood disorders such as depressive symptoms (10–12). To this end, the world health organization (WHO) has recommended that national (mental)-health policies should actively promote well-being, rather than focusing exclusively on the prevention of (mental)-health disorders (13–15). Crucial, however, to the possible use of well-being promotion to target depressive symptom is knowledge on the nature of the association between well-being and depressive symptoms throughout the lifespan.

Traditionally, well-being and depressive symptoms have been considered as opposite ends of a continuum: that low scores on depressive symptoms are considered to be indicative of high levels of well-being and vice versa (16). However, measures of well-being and depressive symptoms are only moderately correlated (between −0.40 and −0.55 in the general population (10–12). This suggests that well-being and depressive symptoms belong to separable but correlated dimensions (16, 17). For example, it is possible to score low on psychiatric problems but not necessarily score high on well-being, or to score high on psychiatric problems and exhibit high levels of well-being (11, 17, 18). Whether well-being and depressive symptoms are two sides of the same coin is still widely debated, and more research is needed to understand the commonalities and specificities underlying this association.

Twin-family studies are important in unraveling phenotypic associations. More specifically, twin studies can be used to examine the role of shared genetic and environmental influences in the relationship between traits (19), and have demonstrated that genetic factors play a substantial role in explaining the observed phenotypic correlation between well-being and depressive symptoms (10, 20–23). For example, genetic influences explain between 33 and 66% of the phenotypic association between well-being and depressive symptoms in adolescents (10, 20). Twin studies on the association between well-being and depressive symptoms in adult populations yield similar bivariate heritability estimates ranging between 40 and 74% (21–23). Additionally, these studies report genetic correlations (a quantification of the extent to which two traits are influenced by the same genes) between well-being and depression in the range of −0.55 to −0.79, which is consistent with a recent large scale genome-wide association study that reported a genetic correlation of −0.75 (24).

While these findings extend our knowledge on the association between well-being and depressive symptoms, some limitations exist. First, the current literature is limited to adolescent (10, 20) and adult samples (17, 21–23). Given the growing interest in well-being promotion across the lifespan (14, 15), and the interest of policy makers in early-years interventions to reduce childhood risks (25), it is important to extend these studies to younger ages. Second, studies thus far focused on a specific age group, namely either adolescence or adulthood. As a result, the current literature lacks a perspective on the well-being—depressive symptom relationship throughout the lifespan. Given the complex development of depressive symptoms from childhood into adulthood (26), the changing genetic, and environmental architecture of depressive symptoms over age (27), and the genetic stability of well-being (28, 29), it is possible that contributions of genetic or environmental factors to the relationship between well-being and depressive symptoms vary over the lifespan. Therefore, a genetically informed study from childhood to adulthood is required to provide the necessary insights into the sources of phenotypic overlap between well-being and depression and thereby detect possible vulnerable but also malleable periods.

In sum, the aim of the present study is to evaluate the contributions of genetic and environmental factors to the association between well-being and depressive symptoms over the lifespan using an informative twin-design. Doing so, we provide better insight into the etiological underpinnings of the association, possibly articulating more targeted models of well-being promotion.

## Methods

### Sample

Participants were registered with the Netherlands Twin Registry (NTR), which consists of the Young NTR (YNTR) (30) and the Adult NTR (ANTR) (31). Subject recruitment is ongoing and is based on voluntary basis, for example, through the website of the register (https://www.tweelingenregister.org/) and through the “Dutch association for parents of multiples,” NVOM (https://www.nvom.nl/). The YNTR twins were registered with the NTR as newborns and were followed throughout childhood and adolescence. Parents completed questionnaires concerning their children when the children were approximately 1, 2, 3, 5, 7, 9/10, and 12 years old. The parents were asked for consent to send their children self-report surveys from age 14 onwards. Given parental consent, twins and their non-twin-siblings received an online or a paper self-report survey when they were 14, 16, or 18 years old. When young twins reached the age of 18, they were enrolled in the ANTR. The ANTR includes adolescence and adults, who were recruited through city councils and by other means (31), and who receive self-report questionnaires every 2–3 years. Participants are allowed to unsubscribe at any moment.

The current study included all twins between the ages 7 and 99 years, with data on either or both well-being and depressive symptoms. Specifically, we included twins from the YNTR at age 7, 9/10, 12, 14, and 16 years old. Data from several age groups are collapsed, because of the relatively recent addition of well-being questions to the survey studies of the NTR. ANTR participants were divided in young adults (age-range 18–27 years) and adults (>27 years). We decided on the age bracket of 27 years for the following reasons. First, this cut-off is in line with earlier twin studies involving comparable traits (27). Second, in the Dutch population, the late twenties are characterized by new “live events” such as fulltime working live, considering marriage and having children (32, 33). Third, in order to obtain reliable estimates a minimum sample size is essential. With a cut-off at age 27 reasonable sample sizes are obtained in each age group. The total dataset comprised 42,427 twins, including 16,089 monozygotic (MZ) and 26,338 same-sex and opposite-sex dizygotic (DZ) twins. The majority (54.6%) participated in more than one NTR survey study; with 16.9% taking part 3 times or more. Participants came from all regions of the Netherlands, both rural and urban areas, and were primarily Caucasian. For a detailed overview of included participants in the different age bins for well-being and depressive symptoms respectively see Supplementary Table 1 and Supplementary Figure 1.

### Measures

Maternal and self-report ratings based on the Cantril ladder (34) were analyzed for children and (young) adults. The ladder has 10 steps where step 10 indicates the best possible life, and step 1 indicates the worst possible life. Participants were asked to indicate well-being by choosing the step which corresponded to the evaluation of their general well-being (self-ratings, age 14 >) or the general well-being of their child (maternal ratings, age 7–12). In our previous work (35), we report on moderate to strong positive correlations between the Cantril ladder and other measures of well-being [see also (2, 36)]. This measure is frequently used [e.g., see (4)]. Test-retest analyses showed test-retest correlations between 0.66 and 0.70; (37) as well as a substantial degree of concurrent validity with multi-item well-being scales [correlation between 0.62 and 0.64; (38)].

Depressive symptoms were assessed by the “Anxious/Depressed” subscale of the age-appropriate survey of the Achenbach's System of Empirical Based Assessment (ASEBA). At ages 7, 9/10, and 12, maternal reports on the Child Behavior Checklist [CBCL/4-18; (39)] were collected. Participants in the age range 14–16 years, completed the Youth Self Report [YSR; (40)] and adults completed the Adult Self Report [ASR; (41)]. The instruments were designed to measure comparable constructs over the ages. All instruments collect symptom information on a 3-point scale, “*Not true*,” “*somewhat true or sometimes true*,” “*very or often* true.” Reliability and validity tests of the “Anxious/Depressed” subscale revealed test-retest correlations in the range of 0.74–0.82 with a Cronbach's alpha of 0.84 (39, 41).

## Strategy of analyses

### Descriptive statistics and phenotypic correlations

Descriptive statistics and phenotypic correlations between well-being and depressive symptoms were calculated in R (42). Furthermore, we tested for main effects of sex and age on the two phenotypes.

### Bivariate genetic modeling

We applied structural equation modeling to the twin data to estimate contributions of genetic and environmental factors to the phenotypic variance of well-being and depressive symptoms and to their phenotypic covariance. The classical twin design exploits the fact that monozygotic (MZ) twins are genetically identical and dizygotic twins (DZ) share on average 50% of their genetic material to estimate genetic, shared, and unshared environmental variance components. We can estimate additive genetic (A), shared environmental (C), non-shared environmental (E), and dominance genetic (D) components of variance and covariance. As C and D both increase the DZ correlation relative to the AE model they cannot be identified simultaneous in the presence of A and E and will therefore be modeled separately (43).

The depressive symptoms scores were strongly skewed (L-shaped distribution). Such non-normality may bias estimates of environmental influences on the phenotype (44). Thus, we categorized the depressive symptoms data into three (low, middle, high) groups, and analyzed it as an ordinal variable, assuming an underlying liability with a normal distribution and with two thresholds (45). The variance in the liability is subject to the decomposition into genetic and environmental components. The two-threshold model determines the prevalence of the low, middle, and high depressive symptoms scores. The well-being measure was modestly skewed to the right, but largely characterized by a bell-shaped curve and was analyzed as a continuous variable.

The bivariate genetic analyses were performed in OpenMx (46). Within each age-group we estimated the summary statistics separately in the MZmales (MZM), DZmales (DZM), MZfemales (MZF), DZfemales (DZF), and Dizyogitic Opposite Sex (DOS) twin pairs. We estimated the thresholds of the ordinal variables separately in males and females. Sex differences in well-being mean scores and prevalence in depressive symptoms were analyzed by testing whether the means (well-being) or thresholds (depressive symptoms) of males and females could be constrained to be equal.

We used the log-likelihood ratio test to evaluate the significance of parameter estimates. This involves fitting the model with and without the constraints of interest, and basing the test statistic on difference in minus twice the differences in loglikelihood of the models, i.e., the log likelihood ratio (LLR). If the constraints of interest are tenable, this statistic follows a central χ^2^ distribution with degrees of freedom (*df*) equal to difference in number of free parameters in the two models. The more parsimonious model (i.e., including the constraints of interest) is rejected if the LLR statistic exceeds the value of *p* < 0.005 (47). If this is not the case, the more parsimonious model is retained.

**Figure 1:**
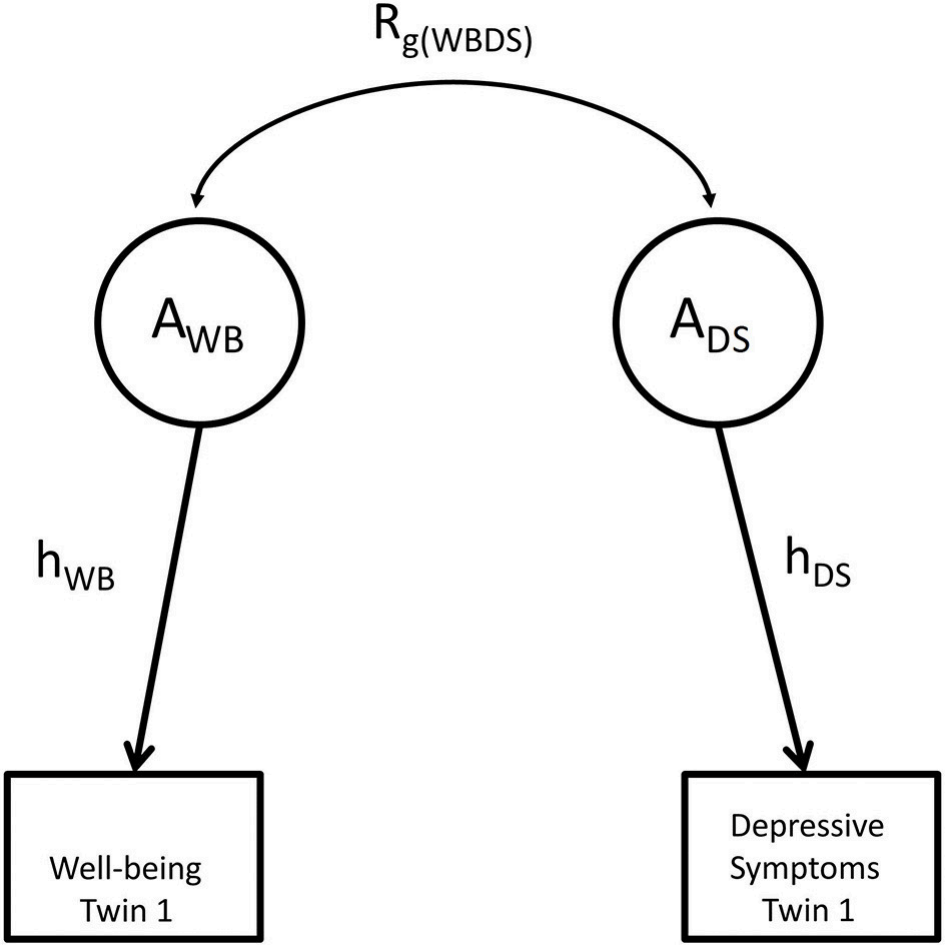
The relationship between shared heritability and genetic correlation. _g_ represents genetic factors influencing well-being and depression; R_g(WBDS)_ represents the genetic correlation between both phenotypes. Shared heritability equals the path r_g(WBDEP)_h_WB_h_DEP_, where h_WB_ equals the square root of univariate heritability for well-being and h_DEP_ equals the square root of the univariate heritability for depressive symptoms.

We estimated genetic and environmental contributions to the bivariate phenotypic covariance matrix by decomposing the phenotypic covariance matrix into (2 × 2) A, C, and E covariance matrices, or (2 × 2) A, D, and E covariance matrices. Bivariate heritability is a function of the heritability of the two traits and the genetic correlation (see Figure 1). Bivariate heritability tell us what the contribution is of genetic factors to the phenotypic association of well-being and depressive symptoms. The genetic correlation quantifies the extent to which two traits are influenced by the same genes regardless of the magnitude of the contribution of genes (the bivariate heritability) to the phenotypic variance of the traits. We parameterized the covariance matrices using a bivariate Cholesky decomposition (46). We first considered the full ACE or ADE bivariate models, and then fitted reduced models, in which we tested various parameters (e.g., the variance components due to shared environmental or dominance effects). Having established the best fitting bivariate models based on log likelihood tests, we calculated 95% confident intervals of all free parameters in the model of choice.

## Results

### Descriptives and phenotypic correlations

Means, standard deviations, and thresholds of males and females in all age-groups are provided in Table 1. The means of both maternal and self-reported ratings of depressive symptoms were significantly higher in females (*p* < 0.005), with largest effect size observed at age 16 (Cohen's *d* = −0.62). Sex differences in well-being scores were observed and strongest in adolescence at age 14. At this age, females reported lower levels of well-being (*p* < 0.005, Cohen's *d* = 0.19), but the effect was smaller compared to sex differences in depressive symptoms scores. In general, depressive symptoms scores tended to increase with age, whereas well-being scores were more stable, but it shows a decrease from adolescence onwards in both sexes (Supplementary Table 1).

**Table 1:** Mean and standard deviation for the raw data for all age groups, as well as the thresholds for the liability distribution and the percentages of twins in the three groups.

	**Age 7 Mean (*sd*)**	**Age 10 Mean (*sd*)**	**Age 12 Mean (*sd*)**	**Age 14 Mean (*sd*)**	**Age 16 Mean (*sd*)**	**Age 18–27 Mean (*sd*)**	**Age 27–99 Mean (*sd*)**
**MALES**							
**Mean**							
Well-being	8.39 (0.98)	8.27 (1.05)	8.22 (1.12)	8.06 (1.03)	7.82 (1.03)	7.57 (1.10)	7.76 (1.01)
Depressive symptoms	2.19 (2.58)	2.26 (2.79)	1.99 (2.69)	2.57 (2.83)	2.63 (2.80)	3.54 (3.81)	2.77 (3.16)
**CATEGORIES**							
**Depressive symptoms**							
Threshold 1	0.05	0.07	−0.31	0.31	0.26	−0.46	−0.20
Threshold 2	0.65	0.69	0.42	0.90	0.63	0.38	0.42
Low	52.0%	52.9%	37.7%	62.3%	60.1%	32.3%	42.0%
Middle	25.8%	24.4%	33.7%	18.3%	26.6%	35.3%	33.6%
High	22.2%	22.7%	28.5%	19.4%	13.3%	32.4%	24.4%
**FEMALES**							
**Mean**							
Well-being	8,42 (0.95)	8.37 (0.98)	8.27 (1.16)	7.85 (1.16)	7.63 (1.13)	7.51 (1.09)	7.67 (1.12)
Depressive symptoms	2.36 (2.61)	2.44 (2.90)	2.21 (2.76)	4.51 (3.90)	4.91 (4.08)	5.25 (4.55)	4.28 (3.86)
**CATEGORIES**							
**Depressive symptoms**							
Threshold 1	−0.04	−0.01	−0.45	−0.34	−0.46	−0.92	−0.71
Threshold 2	0.59	0.66	0.38	0.75	0.46	0.47	0.4
Low	48.2%	49.6%	32.7%	36.6%	32.3%	17.7%	23.6%
Middle	27.8%	25.6%	35.3%	22.5%	32.2%	32.0%	34.6%
High	24.0%	24.7%	32.0%	40.9%	35.5%	50.3%	41.8%

Well-being and depressive symptoms are significantly correlated and the correlation increases with age, ranging from −0.34 during childhood to −0.49 in adulthood (see Table 2).

**Table 2:** Phenotypic correlations, twin correlations and cross-twin cross-trait correlations for well-being and depressive symptoms.

	**Phenotypic**	**MZ**		**DZ**	
	**WB**	**WB**	**DS**	**WB**	**DS**
WB y7	1	0.85 (0.83, 0.87)		0.66 (0.62, 0.69)	
DS y7	−0.34 (−0.38 to −0.28)	−0.30 (−0.37, −0.22)	0.71 (0.68, 0.73)	−0.36 (−0.41, −0,30)	0.46 (0.44, 0.48)
WB y10	1	0.79 (0.76, 0.81)		0.62 (0.59, 0.65)	
DS y10	−0.41 (−0.45 to −0.35)	−0.43 (−0.48, −0.35)	0.71 (0.68, 0.73)	−0.45 (−0.50, −0.41)	0.45 (0.42, 0.48)
WB y12	1	0.83 (0.81, 0.85)		0.63 (0.60, 0.65)	
DS y12	−0.39 (−0.43 to −0.34)	−0.34 (−0.40, −0.28)	0.70 (0.67, 0.72)	−0.39 (−0.43, −0.35)	0.46 (0.43, 0.48)
WB y14	1	0.46 (0.42, 0.50)		0.25 (0.21, 0.29)	
DS y14	−0.44 (−0.48 to −0.38)	−0.38 (−0.43, −0.33)	0.60 (0.55, 0.64)	−0.38 (−0.42, −0.34)	0.28 (0.22, 0.33)
WB y16	1	0.47 (0.42, 0.52)		0.21 (0.16, 0.26)	
DS y16	−0.47 (−0.50 to −0.40)	−0.44 (−0.50, −0.39)	0.52 (0.46, 0.57)	−0.40 (−0.44, −0.35)	0.23 (0.16, 0.29)
WB y18–27	1	0.42 (0.37, 0.48)		0.16 (0.11, 0.22)	
DS y18–27	−0.57 (−0.59 to −0.50)	−0.51 (−0.56, −0.45)	0.56 (0.50, 0.62)	−0.53 (−0.58, −0.49)	0.28 (0.21, 0.35)
WB >27y	1	0.30 (0.25, 0.35)		0.11 (0.04, 0.19)	
DS > 27y	−0.49 (−0.54 to −0.45)	−0.50 (−0.55, −0.45)	0.49 (0.43, 0.54)	−0.56 (−0.60, −0.50)	0.15 (0.06, 0.23)

### Twin correlations

MZ and DZ twin correlations and cross-twin-cross trait correlations for each age-group are displayed in Table 2. In childhood (i.e., age < 14 years), the MZ correlations of both phenotypes were lower than twice the DZ correlations indicating the contribution of additive genetic (A) shared environment (C) and unique environmental (E) effects to the phenotypes. In adolescence and adulthood, both MZ and DZ correlations decreased, resulting in MZ correlations being larger than twice the DZ correlations, which suggests a role for dominant genetic effects (D) besides additive genetic effects. In these age-groups, an ADE model was fitted to the data to establish the presence of dominance genetic influences. Twin correlations and cross-twin cross trait correlations for each of the five zygosity and age-groups are summarized in Supplementary Table 2. The MZ correlations were always higher than the DZ correlations, indicating that genetic effects play a role in explaining individual differences in well-being and depressive symptoms. Sex differences were investigated by constraining the correlations of MZ males to MZ females and DZ males to DZ females. We observed sex differences in the correlation for well-being at ages 7, 14, and 18–27, and differences for depressive symptoms at age 14. The largest difference in twin correlations was observed at age 7 for well-being between MZ males (*r* = 0.82) and MZ females (*r* = 0.89). However, since the differences were relatively rare and small (largest Cohen's *d* is 0.3; Supplementary Table 3), we decided not to model sex specific effects in the variance decomposition. However, we did retain sex differences in the means and thresholds, allowing for a main effect of sex.

### Bivariate genetic analyses

The proportions of phenotypic variance of well-being and depressive symptoms attributable to genetic variance, the heritability (h^2^), and environmental variance effects (i.e., c^2^ and e^2^) are displayed in Table 3 and Supplementary Figure 2. For both well-being and depressive symptoms, a substantial amount of the phenotypic variances was due to additive genetic effects. For well-being, genetic effects explained 31–47% of the phenotypic variation, while for depressive symptoms estimates were between 49 and 61%. Supplementary Table 4 shows the result of the Cholesky decompositions, illustrating the full model and sub-models that were tested. Models in bold are judged to provide the best model fit.

**Table 3:** Standardized estimates (95 % CI) for additive genetic, shared and non-shared environmental influences on well-being and depressive symptoms and their covariance based on the best fitting model.

	**A**		**C**		**E**	
	**WB**	**DS**	**WB**	**DS**	**WB**	**DS**
WB y7	0.43 (0.37–0.49)		0.43 (0.37–0.49)		0.13 (0.12–0.16)	
DS y7	0.49 (0.29–0.70)	0.49 (0.29–0.70)	0.29 (0.10–0.48)	0.20 (0.15–0.25)	0.21 (0.16–0.27)	0.29 (0.27–0.31)
WB y10	0.40 (0.34–0.47)		0.41 (0.35–0.46)		0.20 (0.17–0.21)	
DS y10	0.41 (0.26–0.57)	0.53 (0.46–0.60)	0.30 (0.17–0.44)	0.18 (0.12–0.23)	0.28 (0.23 −0.34)	0.28 (0.27–0.31)
WB y12	0.36 (0.31–0.41)		0.46 (0.41–0.50)		0.18 (0.17–0.20)	
DS y12	0.49 (0.32–0.66)	0.50 (0.43–0.58)	0.23 (0.08–0.38)	0.19 (0.14–0.26)	0.28 (0.22–0.34)	0.30 (0.27–0.32)
WB y14	0.47 (0.43–0.50)		–		0.53 (0.50–0.57)	
DS y14	0.77 (0.70–0.84)	0.60 (0.57–0.65)	–	–	0.23 (0.16–0.30)	0.39 (0.35–0.43)
WB y16	0.45 (0.32–0.50)		–		0.55 (0.51–0.59)	
DS y16	0.68 (0.47–0.78)	0.53 (0.42–0.58)	–	–	0.32 (0.25–0.40)	0.47 (0.42–0.52)
WB y18–27	0.42 (0.37–0.47)		–		0.58 (0.53–0.63)	
DS y18–27	0.60 (0.52–0.67)	0.57 (0.52–0.62)	–	–	0.40 (0.33–0.48)	0.43 (0.38–0.48)
WB > 27y	0.31 (0.18–0.36)		–		0.69 (0.64–0.75)	
DS > 27y	0.46 (0.36–0.58)	0.50 (0.42–0.55)	–	–	0.54 (0.46–0.62)	0.50 (0.45–0.55)

**Figure 2:**
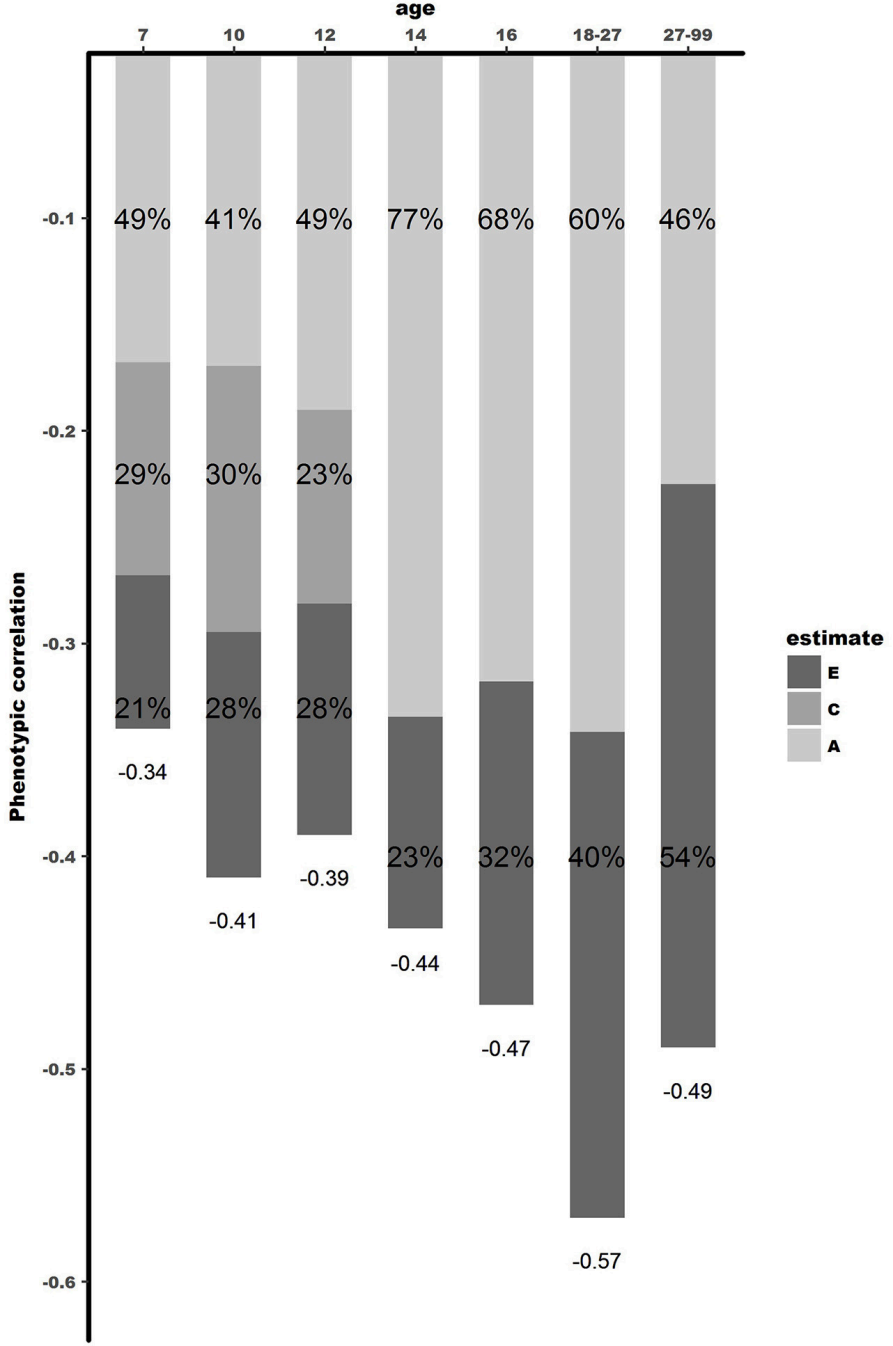
Dissection of phenotypic correlation between well-being and depressive symptoms over the lifespan by shared genetic -and environmental effects. A is the proportion of phenotypic correlation explained by shared genetic effects, C by shared environmental effects, and E by unique environmental effects.

The bivariate Cholesky decomposition provide the decomposition of the variance of the two phenotypes and the decomposition of their covariance into genetic and environmental components (Supplementary Table 4). The results are presented in Figure 2 and Table 3. In childhood, additive genetic and shared environmental effects contribute significantly to the phenotypic correlations. The bivariate heritability ranged from 41 to 49% and bivariate shared environmental effects ranged from 23 to 30%. In adolescence and young adults, additive genetic and non-shared environmental factors contribute largely to the phenotypic correlation, with genetic effects explaining a slightly larger proportion of the phenotypic correlation (range 60–77%). In adults over 27 years, non-shared environmental effects explained 54% of the phenotypic correlation, with the rest explained by additive genetic effects.

**Figure 3:**
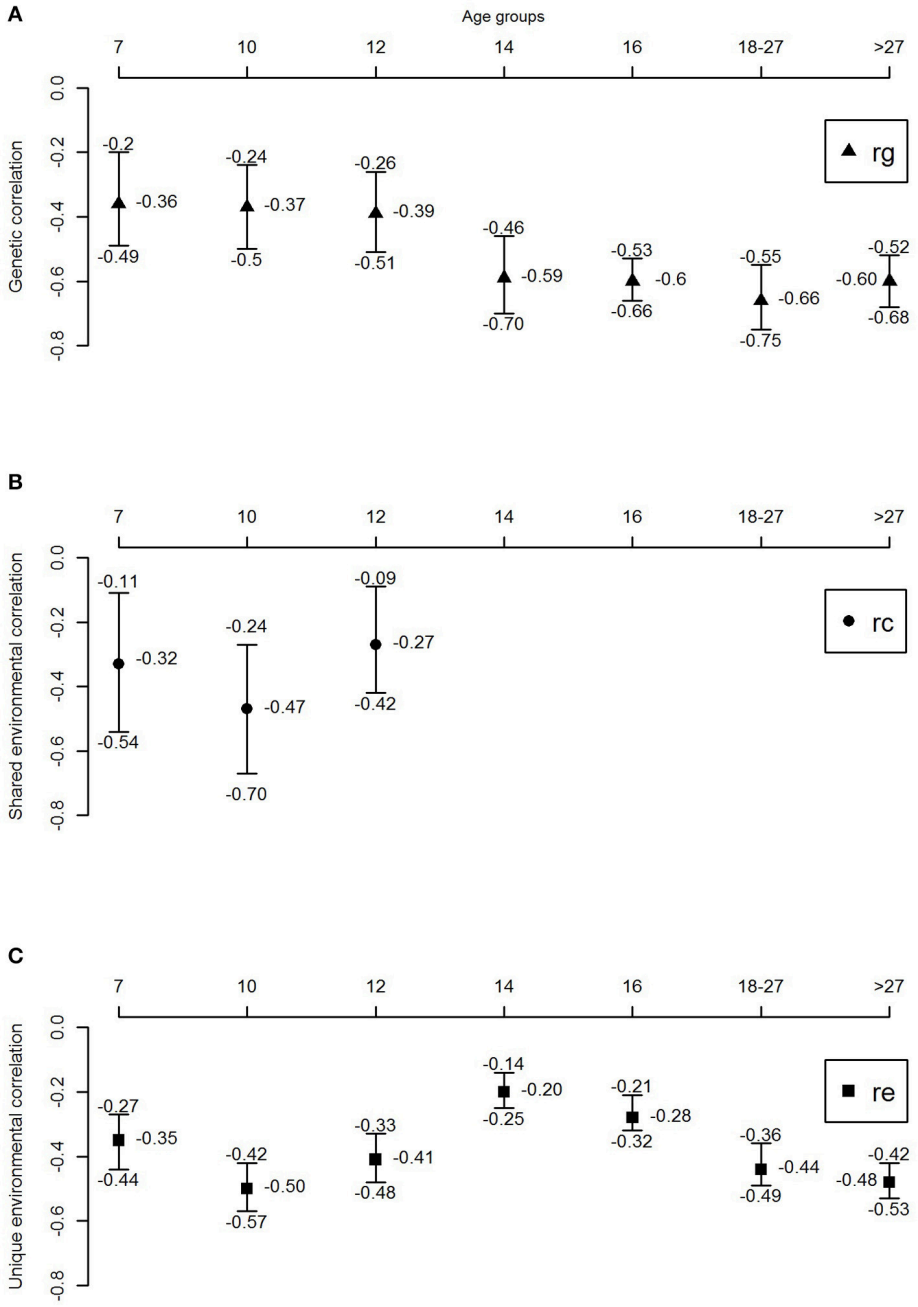
Genetic and environmental correlations between well-being and depressive symptoms over the lifespan. **(A)** Genetic correlation, **(B)** shared environmental correlation, and **(C)** unique environmental correlation.

Figure 3 and Table 4 shows the genetic correlations (r_G_) and environmental (r_C_/r_E_) correlations between well-being and depressive symptoms for all age-groups. In childhood, we observe moderate genetic and environmental correlations, indicating that, while part of the genetic (correlations ranged between −0.36 and −0.39) and environmental (r_C_ ranged from −0.27 to −0.47 and r_E_ ranging from −0.35 to −0.5) susceptibility to well-being and depressive symptoms overlap, there are substantial trait specific genetic and environmental influences. With increasing age, from adolescence onwards, genetic correlations seems to become more important (range −0.59 to −0.66), while the environmental correlations decrease and become limited to non-shared environmental overlap (range −0.20 to −0.48).

**Table 4:** Genetic, shared environmental and unique environmental correlations with their corresponding 95% confidence intervals.

**Age**	***rg***	***rc***	***re***
7	−0.36 (−0.49 to −0.20)	−0.33 (−0.54 to −0.11)	−0.35 (−0.44 to −0.27)
10	−0.37 (−0.50 to −0.24)	−0.47 (−0.67 to −0.27)	−0.50 (−0.57 to −0.42)
12	−0.39 (−0.51 to −0.26)	−0.27 (−0.42 to −0.09)	−0.41 (−0.48 to −0.33)
14	−0.59 (−0.70 to −0.46)	–	−0.20 (−0.25 to −0.14)
16	−0.60 (−0.66 to −0.53)	–	−0.28 (−0.32 to −0.21)
18-17	−0.66 (−0.75 to −0.55)	–	−0.44 (−0.49 to −0.36)
>27	−0.60 (−0.68 to −0.52)	–	−0.48 (−0.53 to −0.42)

## Discussion

The aim of this study was to gain insight into the etiology of the association between well-being and depressive symptoms across the lifespan. Phenotypic correlations between well-being and depressive symptoms ranged from −0.34 in childhood, to −0.49 in adulthood, with the highest correlations in young adults (−0.57). Bivariate twin models revealed that shared environmental factors play an important role in explaining the relationship between well-being and depressive symptoms in childhood, while in adolescence and adulthood genetic factors become increasingly important.

The results of our study go beyond the available literature in several ways. First, the few twin studies carried out so far focused either on adolescents (10, 20) or adults (17, 21–23). Our study is the first to extend these analyses to middle childhood, investigating the association between well-being and depressive symptoms in a cohort-sequential design for age 7, 9/10, and 12, respectively. Results showed that common environmental factors (ranging between 23 and 30%), unique environmental factors (ranging between 21 and 28%), and genetic factors (ranging between 41 and 49%), explain the phenotypic correlation between well-being and depressive symptoms in middle childhood.

Second, instead of focusing on a specific age group, this study examined the association between well-being and depressive symptoms across the lifespan. This study allowed us to shed light on contributions of genetic and environmental factors at different ages. Remarkably, from childhood to adolescence a stark increase was found in the contribution of genetic factors. In adolescence, and young adults, 60–77% of the phenotypic association was explained by genetic factors, with no influence of the shared environment. When looking at the genetic correlations, indicating to what extend the same group of genes influence different traits, moderate to high genetic correlations (between r_g_ = −0.59 and r_g_ = −0.66) were observed in adolescence, while in childhood environmental correlations are substantial, but genetic correlations small (ranging between r_g_ = −0.36 and r_g_ = −0.39). These results show that environmental factors are important in explaining the relationship between well-being and depressive symptoms in childhood, while in adolescence genetic factors play a more substantial role. In adulthood, unique environmental effects showed to be increasingly important, explaining 54% of the phenotypic correlation (with bivariate heritability of 46%). Genetic correlations were high in adulthood, with r_g_ = −0.60, showing overlap in genetic factors influencing both well-being and depressive symptoms. These results were consistent with the results of Kendler et al. (21), who reported similar genetic and phenotypic correlations. However, the proportion of the phenotypic correlation explained by genetic effects was larger in their study 86%, compared to 46% in our study. A possible explanation might be that the heritability of the latent factor mental well-being in Kendler et al. (21) was substantially higher (72%) than the heritability of our measure of well-being (31%). This is attributable to their assessment of well-being which is modeled with a latent factor allowing to correct more explicitly for measurement error. In our design, not modeling well-being as a latent factor, part of the measurement error falls into the E component instead of the additive genetic component explaining the discrepancy in heritability estimates (48).

Overall, the moderate phenotypic correlations between well-being and depressive symptoms in the present study support the notion that well-being and depressive symptoms could belong to distinct, but correlated, dimensions. However, results of the genetic informative twin design shows that shared genetic effects explain a substantial part of this phenotypic correlation, especially from adolescence onwards. This finding raises the question whether different interventions are needed for promoting well-being and treating depressive symptoms, or whether we can use the promotion of well-being to reduce depressive symptoms. On the one hand, a growing body of literature suggests that, based on the unique environmental influences on both well-being and depressive symptoms, interventions targeting well-being may not necessarily have a direct impact on depressive symptoms (17, 20). On the other hand, empirical studies suggest that improved positive emotions enhance coping skills, weaken physiological effects of negative emotions and diminish relapses in depressed individuals (49–53). Additionally, a recent meta-analysis on the effectiveness of positive psychology interventions, including 51 studies and 4,266 individuals, illustrate that, overall, enhancing well-being with positive psychology interventions significantly decrease depressive symptoms (54). These findings, together with our results, suggest that well-being could be used in future studies as an index of mental health complementing other indices that focus on mental illness.

Still, the question remains if these findings hold for the prevention of depressive symptoms by early screening and well-being promotion. Put differently, can we use measures of well-being to inform us about vulnerability to depression? Benefits of this approach include the low stigma associated with the content of well-being questionnaires compared to depressive symptom screening (i.e., people are more willing to answer questions on their quality of life than on their depressive symptoms), and the possibility of screening those at risk in a timely manner. The relatively strong genetic correlation implies that we can identify individuals characterized by low well-being, and offer them suitable interventions to improve their well-being. Even stronger effect may be anticipated if we consider well-being promotion at a population level. Within epidemiology and somatic medicine, it has been proposed that larger benefits to overall public health are to be expected when the bell curve of mental health in the human population is shifted slightly to the healthy side, the so-called population strategy (55, 56). Specifically, a relative slight increase in the level of well-being of the majority of the population may have a larger preventive effect, than targeting the much smaller group of people at high risk or in the early stages of depressive symptoms.

Future studies should, however, focus on the direction of the relationship between well-being and depressive symptoms to use well-being as a possible candidate for novel approaches to reduce depressive symptoms. Recent methodological developments such as Mendelian randomization (MR) designs (57, 58) together with the availability of large scale molecular genetic data provide additional opportunities to address the process underlying the correlation between well-being and depressive symptoms.

## Limitations

This study has several strengths and weaknesses. First, well-being is a complex phenotype consisting of two well-recognized constructs: Subjective Well-being (SWB) and Psychological Well-being (PWB), shaped by the philosophical concepts of hedonism and eudaimonism, respectively (59). Hedonic well-being is centered around pleasure, or how good a person feels about his or her life, whereas eudaimonic well-being is centered around living well or doing well and the fulfillment of human capacities (60). We recognize that, by using the Cantril ladder, the present study does not capture the complete construct of well-being, but rather focuses on SWB. We are confident however, that our results are representative for SWB as the different questionnaires measuring SWB used in social and behavioral sciences correlate highly with the Cantril ladder, both phenotypically and genetically (35). Second, it is important to keep in mind that high scores on the CBCL, YSR, and ASR “Anxiety and Depressive symptoms” subscales are good predictors of depressive symptoms (61), but are not equivalent to a clinical diagnosis of depression (62). Third, due to highly skewed scores, we analyzed the depressive symptoms data using a threshold model, resulting in lower statistical power compared to an analysis of continuous data (44). However, the parameter estimates in a threshold model are more accurate than in an analysis of continuous data characterized by large skewness (63). Fourth, in adolescence and adulthood, both MZ and DZ correlations decreased, resulting in MZ correlations being larger than twice the DZ correlations, suggesting a role for dominant genetic effects besides additive genetic effects. We recognize that this might be a methodological artifact as a result of the difference between parent rating (e.g., parents phenotype possibly contribute to similar ratings for both twins resulting in shared environmental influences) and self-rating scores (e.g., a twin's own genetic architecture contributing to their own behavior and therefore self-rating scores on well-being and depressive symptoms) (48, 64–66). However, while differences in parent reports and child reports exist, earlier studies have illustrated sufficient agreement between child and parent reports on children's quality of life (67). Fifth, earlier research (68) has postulated that a U-shaped pattern of well-being mean-scores over time exist. It is important to note that the method applied in this study focuses on variance decomposition, rather than mean comparison. Additional analyses specifically exploring the mean of well-being over time did not yield a U-shaped pattern. Therefore we believe this does not influence the results presented in our paper.

## Future recommendations

As the genetic and environmental factors explaining the relation between well-being and depressive symptoms differ between the included age-bins, future studies are needed to study the etiology of the relationship between well-being and depressive symptoms in more depth. Longitudinal twin designs such as the genetic simplex model or common factor model (with or without age specific influences) allow for estimation of the stability of the effects of genetic and environmental factors over time, and show to what extent genetic innovation come into play. Additionally, future studies are recommended to investigate key biological and environmental factors of relevance to well-being and depressive symptoms. For example, Routledge et al. (69) investigated the link between well-being, depression and cognitive functioning (and their genetic and environmental overlap). They illustrated some differentiation, with well-being in some cases related to specific cognitive functions independent of depression while for other cognitive functions they showed an overlap between well-being and depression. Additionally, the first genetic variants associated with both well-being and depressive symptoms are recently identified (24). With the increasing availability of large-scale genetic data, it would be interesting to study whether different genetic variants are associated with well-being over age and whether these variants have a protective effect on the development of depressive symptoms. Finally, further research should not isolate genetic and environmental influences and preferably apply multi-layer designs incorporate both aspects explaining the underlying etiology of well-being and depressive symptoms. Finally, although this study is about variance decomposition with relatively small sex differences we observed larger sex differences in mean scores especially from adolescence onwards. Future studies should focus on the origin of these differences especially in (pre)-clinical settings. Furthermore, larger studies are needed to investigate sex-differences in variance components as the presence or absence is still inconclusive [see also review (28)].

## Conclusion

In the present study we dissected the association between well-being and depressive symptoms from childhood to adulthood. We confirmed that well-being and depressive symptoms correlate moderately across the lifespan. Importantly, shared environmental factors play an important role in explaining the relationship between well-being and depressive symptoms in childhood. However, from adolescence onward, we found evidence for the prominence of shared genetic effects, with genetic factors explaining a substantial part of the phenotypic correlation from adolescence onward. Therewith, this study provided more insights into the etiological underpinnings of well-being and depressive symptoms, possibly allowing to articulate better strategies for health promotion and resource allocation in the future.

## Data availability

Datasets used in the present studies are available on request to any qualified researcher.

## Research involving human participants

All procedures performed in studies involving human participants were in accordance with the ethical standards of the institutional and/or national research committee and with the 1964 Helsinki declaration and its later amendments and comparable ethical standards.

## Ethics statement

Data collection was approved by the Central Ethics Committee on Research Involving Human Subjects of the VU University Medical Centre Amsterdam, An Institutional Review Board certified by the US Office of Human Research Protections (IRB number IRB00002991 under Federal-wide Assurance- FWA00017598; IRB/institute codes, NTR 03-180).

## Informed consent

Informed consent was obtained from all individual participants included in the study.

## Author contributions

BB wrote the manuscript, performed the main analyses and designed all (supplementary) figures and tables. YW wrote the manuscript and helped with the analyses. MB supervised the project, curated the data and wrote the manuscript. DB supervised the project and curate the data, CvB, LL, and GW were involved in data collection and data curation. CD helped with the analyses and writing.

### Conflict of interest statement

The authors declare that the research was conducted in the absence of any commercial or financial relationships that could be construed as a potential conflict of interest. The reviewer EC and handling Editor declared their shared affiliation.

## Supplementary material

Supplementary Figure 1 Overview of the survey collection.

Supplementary Figure 2 Proportion of phenotypic variance of well-being and depressive symptoms explained over the lifespan by genetic, shared environmental, and unique environmental effects. h^2^ represents the heritability, c^2^ represents shared environmental influences, and e^2^ represents unique environmental influences.

Supplementary Table 1 Sample size, mean scores and standard deviations for well-being and depression over the lifespan separated by age bin and gender. Significance (*p*) and effect size (cohen's D) of mean differences are provided.

Supplementary Table 2 Twin correlations and cross-trait-cross-twin correlations for well-being and depressive symptoms over the lifespan for all five zygosity groups.

Supplementary Table 3 MZmale/MZfemale and DZmale/DZfemale twin correlation differences and corresponding effect sizes.

Supplementary Table 4 Model fitting results explaining the relationship between well-being and depressive symptoms. Bold represents the best fitting model.
