# Supplementary figures and images for: Unraveling the Genetic and Environmental Relationship Between Well-Being and Depressive Symptoms Throughout the Lifespan
Source: Front Psychiatry. 2018 Jun 14;9:261. doi: 10.3389/fpsyt.2018.00261 (PMC6010548; doi:10.3389/fpsyt.2018.00261)

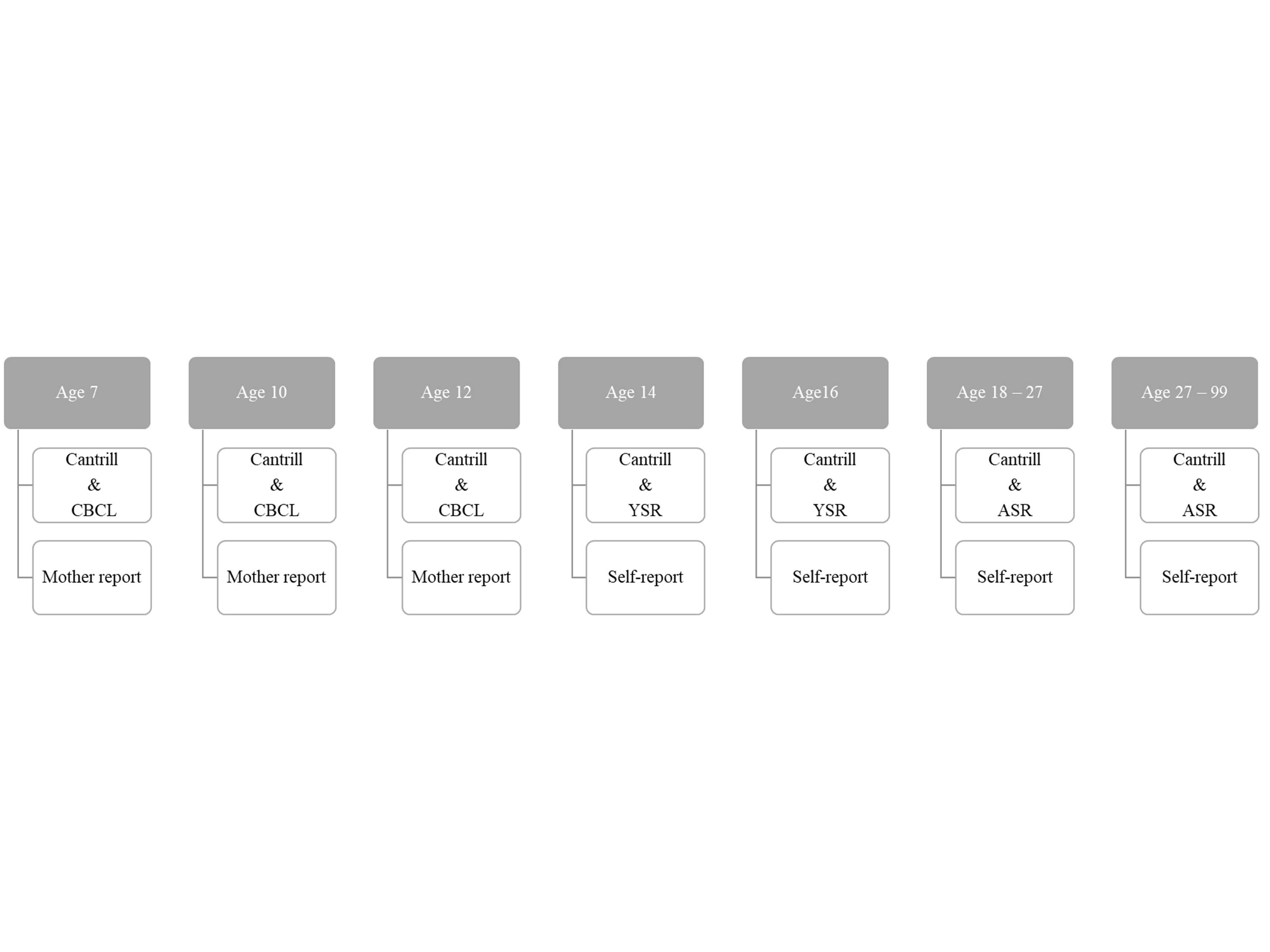

Supplement: Supplementary Figure 1 — Overview of the survey collection. [file Image_1.JPEG]

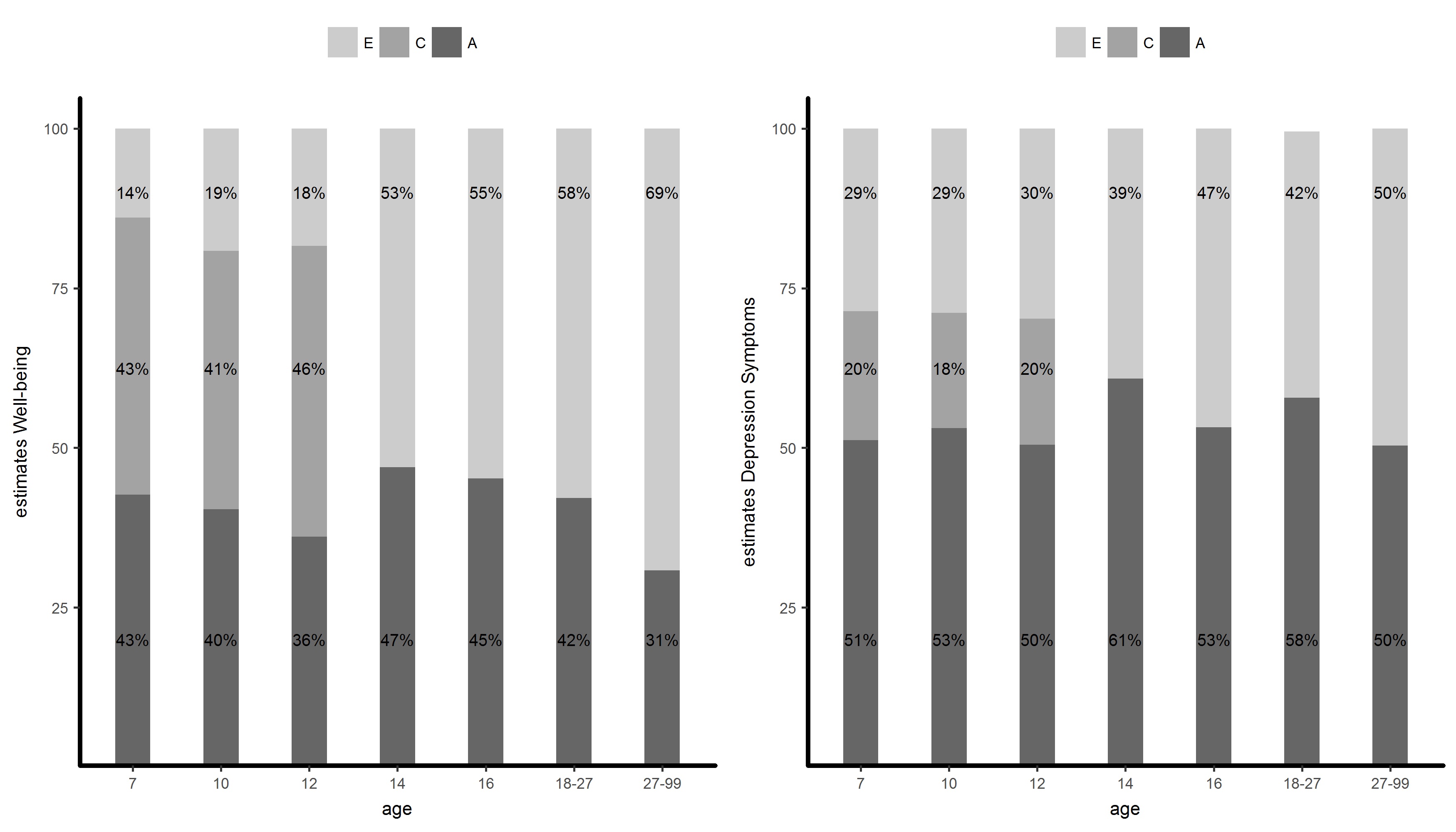

Supplement: Supplementary Figure 2 — Proportion of phenotypic variance of well-being and depressive symptoms explained over the lifespan by genetic, shared environmental, and unique environmental effects. h2 represents the heritability, c2 represents shared environmental influences, and e2 represents unique environmental influences. [file Image_2.JPEG]
