# Supplementary material for: Unraveling the Genetic and Environmental Relationship Between Well-Being and Depressive Symptoms Throughout the Lifespan
Source: Front Psychiatry. 2018 Jun 14;9:261. doi: 10.3389/fpsyt.2018.00261 (PMC6010548; doi:10.3389/fpsyt.2018.00261)
Supplement: Supplementary Table 1 — Sample size, mean scores and standard deviations for well-being and depression over the lifespan separated by age bin and gender. Significance (p) and effect size (cohen's D) of mean differences are provided. [file Table_1.DOCX]

**SI.** Sample size, mean scores and standard deviations for well-being and depression over the lifespan separated by age bin and gender. Significance (*p*) and effect size (cohen’s D) of mean differences are provided. ♂ = male and ♀ = female

| **Well-being** | |  |  |  |  |  |  |  | **Depressive Symptoms** | | |  |  |  |  |
| --- | --- | --- | --- | --- | --- | --- | --- | --- | --- | --- | --- | --- | --- | --- | --- |
| **Age** | ***N*** | ***Mean*** | ***SD*** | **df** | ***t*** | ***p*** | **cohen's d** | | ***N*** | ***Mean*** | ***SD*** | **df** | ***t*** | ***p*** | **cohen's d** |
| 7 ♂ | 2072 | 8.39 | 0.98 | 4035 | -0.74 | 0.09 | -0.02 |  | 12235 | 2.19 | 2.58 | 24572 | -5.124 | 0.014 | -0.07 |
| 7 ♀ | 1965 | 8.42 | 0.95 |  |  |  |  |  | 12339 | 2.36 | 2.61 |  |  |  |  |
| 10 ♂ | 2699 | 8.27 | 1.05 | 5244 | -3.53 | 0.12 | -0.11 |  | 9652 | 2.26 | 2.79 | 19526 | -4.316 | 0.002 | -0.06 |
| 10 ♀ | 2547 | 8.37 | 0.98 |  |  |  |  |  | 9876 | 2.44 | 2.9 |  |  |  |  |
| 12 ♂ | 3236 | 8.22 | 1.12 | 6438 | -1.75 | 0.704 | -0.04 |  | 8186 | 1.99 | 2.69 | 16596 | -5.325 | < 0.01 | -0.08 |
| 12 ♀ | 3204 | 8.27 | 1.16 |  |  |  |  |  | 8412 | 2.21 | 2.76 |  |  |  |  |
| 14 ♂ | 3759 | 8.06 | 1.03 | 8820 | 8.923 | < 0.01 | 0.19 |  | 3892 | 2.57 | 2.83 | 9141 | -26.388 | < 0.01 | -0.55 |
| 14 ♀ | 5063 | 7.85 | 1.16 |  |  |  |  |  | 5251 | 4.51 | 3.9 |  |  |  |  |
| 16 ♂ | 2488 | 7.82 | 1.03 | 6103 | 6.58 | < 0.01 | 0.17 |  | 2696 | 2.63 | 2.8 | 6612 | -25.33 | < 0.01 | -0.62 |
| 16 ♀ | 3617 | 7.63 | 1.13 |  |  |  |  |  | 3918 | 4.91 | 4.08 |  |  |  |  |
| 18-27 ♂ | 2852 | 7.57 | 1.1 | 8256 | 2.313 | 0.316 | 0.05 |  | 3050 | 3.54 | 3.81 | 8779 | -17.777 | < 0.01 | -0.38 |
| 18-27 ♀ | 5406 | 7.51 | 1.09 |  |  |  |  |  | 5731 | 5.25 | 4.55 |  |  |  |  |
| 27-99 ♂ | 7100 | 7.76 | 1.01 | 17968 | 5.172 | < 0.01 | 0.08 |  | 7991 | 2.77 | 3.16 | 19790 | -28.894 | < 0.01 | -0.41 |
| 27-99 ♀ | 10870 | 7.67 | 1.12 |  |  |  |  |  | 11801 | 4.28 | 3.86 |  |  |  |  |
