# Supplementary material for: Unraveling the Genetic and Environmental Relationship Between Well-Being and Depressive Symptoms Throughout the Lifespan
Source: Front Psychiatry. 2018 Jun 14;9:261. doi: 10.3389/fpsyt.2018.00261 (PMC6010548; doi:10.3389/fpsyt.2018.00261)
Supplement: Supplementary Table 2 — Twin correlations and cross-trait-cross-twin correlations for well-being and depressive symptoms over the lifespan for all five zygosity groups. [file Table_2.DOCX]

**S 2.** Twin correlations and cross-trait-cross-twin correlations for well-being and depression over the lifespan for all five zygosity.

| **Age 7** |  |  |  |  | **Age 10** |  |  |  |
| --- | --- | --- | --- | --- | --- | --- | --- | --- |
| Males |  |  | Females |  | Males |  | Females |  |
|  |  |  |  |  |  |  |  |  |
| MZM |  |  | MZF |  | MZM |  | MZF |  |
|  | WB | DEP | WB | DEP | WB | DEP | WB | DEP |
| WB | 0.81 (0.77, 0.84) |  | 0.89 (0.86-0.91) |  | 0.79 (0.76, -0.83) |  | 0.79 (0.75, 0.82) |  |
| DEP | -0.30 (-0.39, -0.19) | 0.69 (0.66, 0.73) | -0.30 (-0.41, -0.19) | 0.72 (0.68, 0.74) | -0.38 (-0.47, -0.28) | 0.71 (0.67, 0.74) | -0.45 (-0.53, -0.35) | 0.71 (0.67, 0.74) |
|  |  |  |  |  |  |  |  |  |
| DZM |  |  | DZF |  | DZM |  | DZF |  |
|  | WB | DEP | WB | DEP | WB | DEP | WB | DEP |
| WB | 0.61 (0.55, 0.68) |  | 0.67 (0.60, 0.73) |  | 0.60 (0.52, 0.65) |  | 0.64 (0.58, 0.69) |  |
| DEP | -0.38 (-0.48, -0.26) | 0.43 (0.38, 0.48) | -0.37 (-0.48, -0.25) | 0.44 (0.39, 0.49) | -0.42 (-0.51, -0.33) | 0.42 (0.36, 0.48) | -0.45 (-0.54, -0.34) | 0.47 (0.41, 0.53) |
|  |  |  |  |  |  |  |  |  |
| Dos/males females | |  |  |  | Dos/males females |  |  |  |
|  | WB | DEP |  |  | WB | DEP |  |  |
| WB | 0.68 (0.63, 0.72) |  |  |  | 0.63 (0.59, 0.67) |  |  |  |
| DEP | -0.33 (-0.42, -0.25) | 0.49 (0.45-0.52) |  |  | -0.46 (-0.52, -0.40) | 0.45 (0.41, 0.49) |  |  |
|  |  |  |  |  |  |  |  |  |
|  |  |  |  |  |  |  |  |  |
| **Age 12** |  |  |  |  | **Age 14** |  |  |  |
| Males |  |  | Females |  | Males |  | Females |  |
|  |  |  |  |  |  |  |  |  |
| MZM |  |  | MZF |  | MZM |  | MZF |  |
|  | WB | DEP | WB | DEP | WB | DEP | WB | DEP |
| WB | 0.83 (0.80, 0.85) |  | 0.83 (0.80, 0.85) |  | 0.37 (0.29, 0.43) |  | 0.50 (0.45, 0.55) |  |
| DEP | -0.36 (-0.44, -0.27) | 0.71 (0.67, 0.75) | -0.33 (-0.41, -0.24) | 0.69 (0.65, 0.72) | -0.33 (-0.41, -0.24) | 0.47 (0.37, 0.56) | -0.41 (-0.47, -0.34) | 0.66 (0.60, 0.71) |
|  |  |  |  |  |  |  |  |  |
| DZM |  |  | DZF |  | DZM |  | DZF |  |
|  | WB | DEP | WB | DEP | WB | DEP | WB | DEP |
| WB | 0.63 (0.57, 0.67) |  | 0.68 (0.63, 0.72) |  | 0.13 (0.04, 0.21) |  | 0.36 (0.28, 0.42) |  |
| DEP | -0.45 (-0.52, -0.37) | 0.40 (0.34, 0.45) | -0.31 (-0.39, -0.21 | 0.51 (0.46, 0.57) | -0.35 (-0.44, -0.27) | 0.28 (0.16, 0.40) | -0.43 (-0.50, -0.35) | 0.30 (0.20, 0.39) |
|  |  |  |  |  |  |  |  |  |
| Dos/males females | |  |  |  | Dos/males females |  |  |  |
|  | WB | DEP |  |  | WB | DEP |  |  |
| WB | 0.60 (0.56, 0.64) |  |  |  | 0.25 (0.20, 0.31) |  |  |  |
| DEP | -0.41 (-0.47, -0.35) | 0.46 (0.42, 0.50) |  |  | -0.35 (-0.41, -0.29) | 0.26 (0.18, 0.33) |  |  |
|  |  |  |  |  |  |  |  |  |
|  |  |  |  |  |  |  |  |  |
| **Age 16** |  |  |  |  | **Age 18-27** |  |  |  |
| Males |  |  | Females |  | Males |  | Females |  |
|  |  |  |  |  |  |  |  |  |
| MZM |  |  | MZF |  | MZM |  | MZF |  |
|  | WB | DEP | WB | DEP | WB | DEP | WB | DEP |
| WB | 0.48 (0.40, 0.54) |  | 0.46 (0.39, 0.52) |  | 0.55 (0.46, 0.63) |  | 0.37 (0.30, 0.43) |  |
| DEP | -0.39, (-0.48, -0.29) | 0.51 (0.40, 0.60) | -0.48 (-0.55, -0.41) | 0.52 (0.44, 0.59) | -0.49 (-0.58, -0.38) | 0.57 (0.46, 0.66) | -0.52 (-0.58, -0.45) | 0.55 (0.47, 0.62) |
|  |  |  |  |  |  |  |  |  |
| DZM |  |  | DZF |  | DZM |  | DZF |  |
|  | WB | DEP | WB | DEP | WB | DEP | WB | DEP |
| WB | 0.14 (0.02, 0.26) |  | 0.25 (0.15, 0.34) |  | 0.07 (0, 0.21) |  | 0.26 (0.17, 0.34) |  |
| DEP | -0.46 (-0.56, -0.37) | 0.20 (0.04, 0.34) | -0.23 (-0.33, -0.12) | 0.28 (0.17, 0.39) | -0.50 (-0.59, -0.39) | 0.24 (0.07, 0.40) | -0.56 (-0.63, -0.48) | 0.35 (0.24, 0.45) |
|  |  |  |  |  |  |  |  |  |
| Dos/males females | |  |  |  | Dos/males females |  |  |  |
|  | WB | DEP |  |  | WB | DEP |  |  |
| WB | 0.22 (0.14, 0.29) |  |  |  | 0.12 (0.03, 0.20) |  |  |  |
| DEP | -0.14 (-0.22, -0.05) | 0.20 (0.10, 0.29) |  |  | -0.52 (-0.59, -0.45) | 0.24 (0.14, 0.34) |  |  |
|  |  |  |  |  |  |  |  |  |
| **Age 27-99** |  |  |  |  |  |  |  |  |
| Males |  |  |  |  |  |  |  |  |
|  |  |  |  |  |  |  |  |  |
| MZM |  |  | MZF |  |  |  |  |  |
|  | WB | DEP | WB | DEP |  |  |  |  |
| WB | 0.35 (0.24, 0.44) |  | 0.29 (0.22, 0.35) |  |  |  |  |  |
| DEP | -0.48 (-0.56, -0.38) | 0.52 (0.41, 0.62) | -0.50 (-0.56, -0.54) | 0.48 (-0.41, 0.54) |  |  |  |  |
|  |  |  |  |  |  |  |  |  |
| DZM |  |  | DZF |  |  |  |  |  |
|  | WB | DEP | WB | DEP |  |  |  |  |
| WB | 0.12 (0-0.28) |  | 0.14 (0.03, 0.25) |  |  |  |  |  |
| DEP | -0.57 (-0.68, -0.44) | 0.11 (0, 0.32) | -0.54 (-0.61, -0.46) | 0.14 (0.02, 0.26) |  |  |  |  |
|  |  |  |  |  |  |  |  |  |
| Dos/males females | |  |  |  |  |  |  |  |
|  | WB | DEP |  |  |  |  |  |  |
| WB | 0.07 (0, 0.18) |  |  |  |  |  |  |  |
| DEP | -0.56 (-0.63, -0.47) | 0.17 (0.04, 0.30) |  |  |  |  |  |  |
