# Supplementary material for: Unraveling the Genetic and Environmental Relationship Between Well-Being and Depressive Symptoms Throughout the Lifespan
Source: Front Psychiatry. 2018 Jun 14;9:261. doi: 10.3389/fpsyt.2018.00261 (PMC6010548; doi:10.3389/fpsyt.2018.00261)
Supplement: Supplementary Table 3 — MZmale/MZfemale and DZmale/DZfemale twin correlation differences and corresponding effect sizes. [file Table_3.DOCX]

**S 3.** MZmale/MZfemale and DZmale/DZfemale twin correlation differences and corresponding effect sizes.

|  |  |  |  |  |  |  | Well-being | |  |  |  |  |  |
| --- | --- | --- | --- | --- | --- | --- | --- | --- | --- | --- | --- | --- | --- |
| Age | MZM | N | MZF | N | Fishers'Z | Cohen's D |  | DZM | N | DZF | N | Fishers'Z | Cohen's D |
| 7 | 0.81 | 769 | 0.89 | 762 | -5.76 | **-0.29** |  | 0.61 | 697 | 0.67 | 598 | 0.16 | 0.01 |
| 10 | 0.79 | 937 | 0.79 | 912 | 0 | 0 |  | 0.6 | 887 | 0.64 | 760 | -1.31 | -0.06 |
| 12 | 0.83 | 1074 | 0.83 | 1115 | 0 | 0 |  | 0.63 | 1110 | 0.68 | 1033 | -2.03 | -0.09 |
| 14 | 0.37 | 1234 | 0.5 | 1932 | -4.41 | -0.16 |  | 0.13 | 1064 | 0.36 | 1391 | -6.04 | -0.25 |
| 16 | 0.48 | 904 | 0.46 | 1377 | 0.6 | 0.03 |  | 0.14 | 650 | 0.25 | 930 | -2.23 | -0.11 |
| 18-27 | 0.55 | 748 | 0.37 | 1662 | 5.21 | 0.21 |  | 0.07 | 549 | 0.26 | 1082 | -3.73 | -0.18 |
| 27plus | 0.35 | 718 | 0.29 | 1960 | 1.53 | 0.06 |  | 0.12 | 323 | 0.14 | 835 | -0.31 | -0.02 |
|  |  |  |  |  |  |  |  |  |  |  |  |  |  |
|  |  |  |  |  |  |  | Depression | |  |  |  |  |  |
| Age | MZM | N | MZF | N | Fishers'Z | Cohen's D |  | DZM | N | DZF | N | Fishers'Z | Cohen's D |
| 7 | 0.69 | 4206 | 0.72 | 4673 | -2.81 | -0.059 |  | 0.43 | 4142 | 0.44 | 3778 | -0.55 | -0.01 |
| 10 | 0.71 | 3354 | 0.71 | 3819 | 0 | 0 |  | 0.42 | 3122 | 0.47 | 2875 | -2.41 | -0.06 |
| 12 | 0.71 | 2870 | 0.69 | 3245 | 1.53 | 0.04 |  | 0.4 | 2640 | 0.51 | 2497 | -4.98 | -0.14 |
| 14 | 0.47 | 1284 | 0.66 | 2003 | -7.9 | -0.28 |  | 0.28 | 1115 | 0.3 | 1471 | -0.55 | -0.02 |
| 16 | 0.51 | 970 | 0.52 | 1511 | -0.33 | -0.01 |  | 0.2 | 706 | 0.28 | 1021 | -1.73 | -0.08 |
| 18-27 | 0.57 | 791 | 0.55 | 1770 | 0.68 | 0.03 |  | 0.24 | 547 | 0.35 | 1127 | -2.31 | -0.11 |
| 27plus | 0.52 | 777 | 0.48 | 2097 | 1.27 | 0.05 |  | 0.11 | 391 | 0.14 | 985 | -0.51 | -0.03 |
