# Supplementary material for: Unraveling the Genetic and Environmental Relationship Between Well-Being and Depressive Symptoms Throughout the Lifespan
Source: Front Psychiatry. 2018 Jun 14;9:261. doi: 10.3389/fpsyt.2018.00261 (PMC6010548; doi:10.3389/fpsyt.2018.00261)
Supplement: Supplementary Table 4 — Model fitting results explaining the relationship between well-being and depressive symptoms. Bold represents the best fitting model. [file Table_4.DOCX]

**S 4.** Model fitting results explaining the relationship between well-being and depression. Bold represents the best fitting model.

| Age7 |  |  |  |  |  |  |  |  |  |  |
| --- | --- | --- | --- | --- | --- | --- | --- | --- | --- | --- |
| Model |  | -2LL |  | df |  | χ^2^ |  | df |  | *p* |
| **ACE** |  | **57449.441** |  | **28598** |  |  |  | **14** |  |  |
| AE |  | 57636.112 |  | 28601 |  | 186.67057 | | 11 |  | <0.001 |
|  |  |  |  |  |  |  |  |  |  |  |
| Age10 |  |  |  |  |  |  |  |  |  |  |
| Model |  | -2LL |  | df |  | χ^2^ |  | df |  | *p* |
| **ACE** |  | **50859.769** |  | **24761** |  |  |  | **14** |  |  |
| AE |  | 51031.227 |  | 24764 |  | 171.45803 | | 11 |  | <0.001 |
|  |  |  |  |  |  |  |  |  |  |  |
| Age12 |  |  |  |  |  |  |  |  |  |  |
| Model |  | -2LL |  | df |  | χ^2^ |  | df |  | *p* |
| **ACE** |  | **51464.688** |  | **23025** |  |  |  | **14** |  |  |
| AE |  | 51730.210 |  | 23028 |  | 265.52208 | | 11 |  | <0.001 |
|  |  |  |  |  |  |  |  |  |  |  |
| Age14 |  |  |  |  |  |  |  |  |  |  |
| Model |  | -2LL |  | df |  | χ^2^ |  | df |  | *p* |
| ADE |  | 43854.645 |  | 17952 |  |  |  | 14 |  |  |
| **AE** |  | **43856.907** |  | **17955** |  | **2.26** |  | **11** |  | **0.52** |
|  |  |  |  |  |  |  |  |  |  |  |
| Age16 |  |  |  |  |  |  |  |  |  |  |
| Model |  | -2LL |  | df |  | χ^2^ |  | df |  | *p* |
| ADE |  | 31113.213 |  | 12706 |  |  |  | 14 |  |  |
| **AE** |  | **31114.154** |  | **12709** |  | **0.94** |  | **11** |  | **0.82** |
|  |  |  |  |  |  |  |  |  |  |  |
| Age18-27 |  |  |  |  |  |  |  |  |  |  |
| Model |  | -2LL |  | df |  | χ^2^ |  | df |  | *p* |
| ADE |  | 33260.502 |  | 13618 |  |  |  | 14 |  |  |
| **AE** |  | **33265.100** |  | **13621** |  | **4.6** |  | **11** |  | **0.20** |
|  |  |  |  |  |  |  |  |  |  |  |
| Age27-99 |  |  |  |  |  |  |  |  |  |  |
| Model |  | -2LL |  | df |  | χ^2^ |  | df |  | *p* |
| ACE |  | 28508.713 |  | 11739 |  |  |  | 14 |  |  |
| **AE** |  | **28517.726** |  | **11742** |  | **9.01** |  | **11** |  | **0.03** |
|  |  |  |  |  |  |  |  |  |  |  |
